# Portable Sequentially Shifted Excitation Raman Spectroscopy to Examine Historic Powders Enclosed in Glass Vials

**DOI:** 10.3390/s22093560

**Published:** 2022-05-07

**Authors:** Silvia Innocenti, Diego Quintero Balbas, Luca Pezzati, Raffaella Fontana, Jana Striova

**Affiliations:** National Research Council, National Institute of Optics, Largo Enrico Fermi 6, 50125 Firenze, Italy; silvia.innocenti@ino.cnr.it (S.I.); diegoivan.quinterobalbas@ino.cnr.it (D.Q.B.); raffaella.fontana@ino.cnr.it (R.F.); jana.striova@cnr.it (J.S.)

**Keywords:** Raman spectroscopy, sequentially shifted excitation, non-invasive investigation, portable instrumentation, heritage science

## Abstract

Raman spectroscopy (RS) is a powerful non-invasive tool for the characterization of materials. However, the fluorescence effect often hampers the detectability of the relatively weak vibrational Raman signal. Several approaches were exploited to overcome this limit. This work, in particular, evaluates the performance of an in situ portable sequentially shifted excitation (SSE™) Raman spectrometer applied to the examination of artistic historical pigment powders enclosed in glass vials. The explored handheld spectrometer employs a dual, temperature-shifted, 785 nm and 852 nm laser excitation to optimize both spectral coverage and fluorescence subtraction. The study demonstrates the feasibility of the SSE RS approach for non-invasive identification of art materials, and its applicability in complex situations where the examined material cannot be removed from its container. Laboratory measurements using benchtop dispersive micro-Raman spectroscopy at 785 nm are reported for comparison.

## 1. Introduction

Preservation of heritage objects requires an exhaustive knowledge of their material composition. Analytical tools to detect and to identify the components of artworks play a fundamental role in heritage science (HS). Diagnostics of heritage objects has always been an analytical challenge, as they cannot be easily moved to laboratories and, in almost all cases, they cannot be sampled nor damaged. For these reasons, non-invasive, portable techniques are mandatory to obtain the required knowledge. One of the most powerful analytical techniques in the heritage scientist’s portfolio is Raman spectroscopy (RS). The technique has been used in HS for more than 40 years, and nowadays it can be considered a routine test to investigate the material composition of artworks, also at the micrometric scale [1]. An increasing number of portable Raman spectrometers was developed [2,3,4,5] and some are now commercial, even if they are mostly optimized for pharmaceutical applications (quick identification of raw materials). Their major advantages are portability, lightness, compactness, ease of use, and user-friendly control interfaces. Their major limitations are low sensitivity, limited spectral range coverage, high levels of fluorescence, and sensitivity to interference from ambient light and environmental conditions [4]. 

Portable RS is less efficient than its laboratory version, which is also due to the undesired effects of fluorescence by the investigated materials resulting from the population of a sample’s excited state(s) [6,7,8]. This is due to fact that, for a given material, the probability of exciting the Raman scattering is much lower than that of exciting fluorescence [9]. Fluorescence appears then in spectra as a broad background band with an intensity proportional to 1/λ_0_, easily covering the weaker Raman bands, thus limiting RS detection capabilities. While in laboratory RS this can be mitigated by the use of different laser sources, in portable RS the choice of sources is severely limited by the portability constraint, thus other techniques were developed over the years to extract Raman spectra from the fluorescent background, among these are: (i) algorithm-based baseline correction [10]; (ii) specialized sampling optics [11]; (iii) time gating [12]; and (iv) shifted excitation [13]. 

Shifting the wavelength of the excitation source is an effective solution for portable RS, as it can exploit the capabilities of laser diodes, which are possibly the lightest and the smallest laser sources available for RS. There are two main implementation pathways: modulated (or encoded) excitation source RS foresees more than two excitation sources while shifted excitation Raman difference spectroscopy relies on two sets of slightly shifted excitation data obtained by thermal shifting of the diode emission. While fluorescence emission remains practically unchanged, the location of Raman lines shifts as a function of the excitation, thus detection is possible even if the signal to noise ratio is low. 

In 2015, a new handheld Raman spectrometer based on the US patent No. 8,570,507B1 was launched by Bruker Optics with the commercial name of BRAVO. The spectrometer implements sequentially shifted excitation (SSE^TM^) [14], where the diode lasers operate at different temperatures, providing slightly shifted wavelengths, and a proprietary algorithm is used to separate the Raman spectra from the fluorescence background. The limited spectral range that characterized the first BRAVO model (300–3200 cm^−1^) did not allow the detection of the low-wavenumber lines characteristic for certain inorganic pigments, such as Pb-based pigments (litharge, red lead, lead tin yellow (I) and (II) types, massicot, and Naples yellow), iron (III) oxide pigments (e.g., hematite, ochres, earths), vermilion, realgar, and pararealgar, having their main lines below 300 cm^−1^ [15,16].

In this work we tested the performance of a recent BRAVO release, with improved firmware and software, allowing the acquisition of a wider spectral region, including lower frequencies (down to 170 cm^−1^). We applied the BRAVO to a case study on real heritage objects: a historical wooden case with glass vials containing pigments in powders. The test has double significance as the measurements were not only obtained in-situ but also without extracting the powders from the vials, thus adding an extra degree of non-invasiveness. 

To verify the potential of the technique, experimental data obtained in situ were compared with the results obtained in a laboratory on the same samples, using a benchtop dispersive Raman spectrometer operating at 785 nm.

## 2. Materials and Methods

### 2.1. Materials

**Figure 1:**
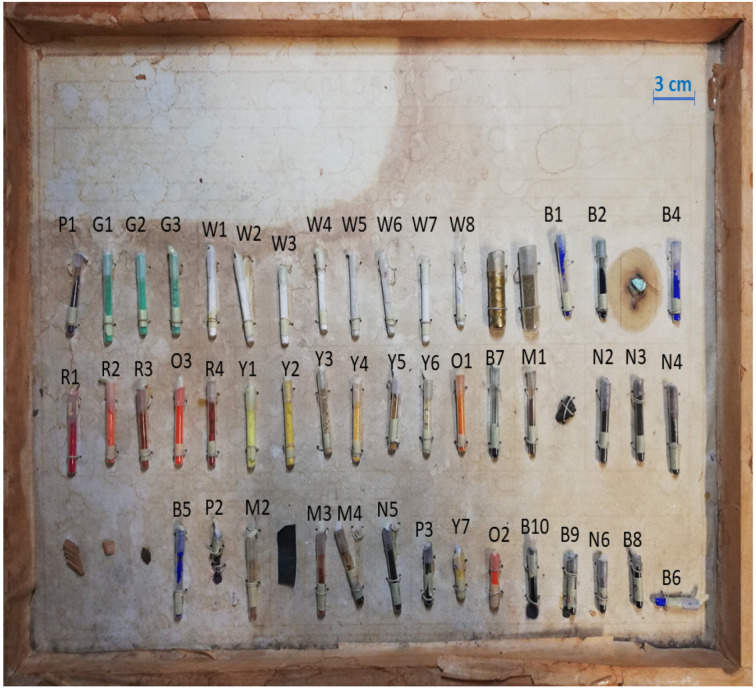
Historical sample-case containing examined pigments mainly enclosed in glass vials.

The examined historical sample-case (52 cm × 36 cm) (Figure 1) consists of a wooden box, entitled *Coloranti. Naturali ed Artificiali*, covered with glued paper sheets. The explanation of the samples contained in it is printed with some handwritten annotations on the paper of the bottom board, unfortunately the text is partially illegible due to ink fading and only some names are visible. The iron hook nailed in the center of the upper wall suggests that the box was meant to be hanged. It comprises 54 samples, of which 48 are fine powder pigments and 6 are driftwood and little stones. The powder samples are enclosed in glass cylindrical vials of three different lengths (1.7 cm, 3 cm, and 4.5 cm), with an external and an internal diameter of, respectively, 0.55 cm and 0.4 cm. The vials, without any identification label, were found closed by cork and glue and attached to the sample-case with threads. The powders were divided into eight hues (blue, yellow, red, green, violet, brown, black, and white). 

The sample-case is located at *Opificio delle Pietre Dure* in Florence (Italy) and in the absence of historical written resource, it belonged most-likely to Augusto Vermehren (Florence, 1888–1978), the son of the painter Otto Vermehren. Augusto was a prominent personality in the field of restoration in the beginning of the 20th century, and he was considered among the founders of scientific restoration [17].

### 2.2. Benchtop and Portable Sequentially Shifted Excitation (pSSE) Raman Spectroscopy

The micro-Raman spectra were acquired with a benchtop Raman confocal microscope (Renishaw inVia) equipped with a Leica DM2700 optical microscope and using a 785 nm excitation diode laser. We performed the measurements in the spectral range 100–3200 cm^−1^, using a grating 1200 L/mm and a thermoelectrically cooled CCD detector (spectral range 400–1060 nm), with a spectral resolution of 1 cm^−1^ per CCD pixel (functional resolution of 3 cm^−1^). The laser power was kept below 7 mW, using a 10s exposure time and 5 or 10 accumulations (Table 1). The cylindrical glass vials were placed horizontally on the motorized microscope stage, with the laser beam focused on the powder inside of the glass vial. Data were collected with a 50x long-working-distance objective (NA Plan = 0.5; theoretical spot size 0.95 μm). Data were processed by Wire5.1 and OriginPro8.5 software. 

**Figure 2:**
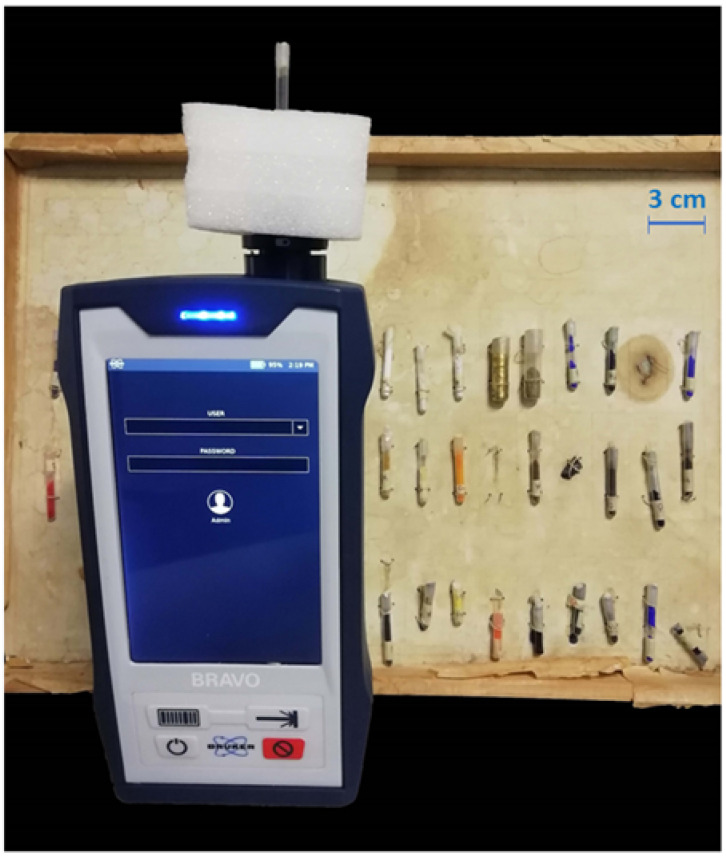
Set-up of pSSE Raman measurement of pigments inside the glass vials placed in the laser beam.

The BRAVO handheld Raman spectrometer (Bruker Optics) has been developed to generate the background-free Raman spectra through the patented sequentially shifted excitation (SSE™). It is equipped with two temperature-controlled diode lasers (DuoLaser™, 785 and 852 nm), comprising Bragg-grating optical feedback. Both laser beams impinge on the sample sequentially in every measurement (to cover a broader spectral range with respect to conventional portable Raman spectrometers), and they are detected using different areas of the charge-coupled device (CCD), providing a 10–12 cm^−1^ spectral resolution. Data can be acquired over a relatively large spectral range, from 170 to 2200 cm^−1^ and from 1200 to 3200 cm^−1^ exploiting, respectively, the 852 nm and the 785 nm lasers. Each laser was temperature-shifted three times over a small wavelength range (about 0.4 nm). As a result, three spectrally shifted raw spectra (by about 6 cm^−1^) were generated by each of the two lasers from which the processed Raman data was extracted by principal component analysis (PCA). The laser power was set automatically, ranging, as measured by a power meter (Thorlabs, PM100D), between 40 and 100 mW, being higher for the 852 nm laser. It was delivered to a laser spot about 100 × 500 μm^2^ wide. Acquisition parameters (detector integration time and number of coadditions) may be set manually only using an external PC (WiFi connected) and the OPUS-IR™ software suite. In this study, the pSSE measurements were acquired with a 0.5–1 s detector integration time and 1–150 coadditions (Table 1). The vials were placed in a vertical position in a tailored polystyrene box to focus the laser beams inside the vial (Figure 2).

### 2.3. Optical Coherence Tomography (OCT)

Spectral-domain OCT (Telesto II, Thorlabs) was used to measure the thickness of the bottom of the glass vials. The instrument operates at 1300 nm (center wavelength) with an axial and a lateral resolution of 5.5 μm (in air) and 13.0 μm, respectively. The data were elaborated with Adobe Photoshop software. The thickness measured by OCT (d_OCT_) was corrected for the refractive index n to achieve the real thickness estimate. Being *n* = 1.52 the refractive index of a generic glass [18], the real glass vials thickness (d_r_) was given by d_r_ = d_OCT_/n [19]. The acquisition field of view (FOV) for the x-z OCT profiles was 10 mm × 2.4 mm with the respective pixel size of 2.5 µm × 2.36 µm. 

## 3. Results

### 3.1. Examination of Glass Containers 

**Figure 3:**
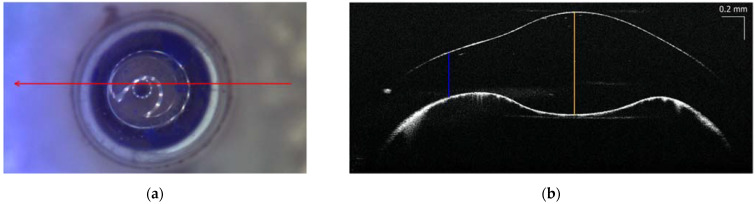
Bottom of the glass vials (upside down): (**a**) visible image—10 mm long red arrow denotes the position of the OCT measurement in b; (**b**) cross-sectional OCT image—the yellow and blue lines indicate the thickness of glass to be, respectively, 0.9 mm and 0.4 mm.

As a first step, the glass vials were inspected with the SD-OCT to determine the thickness of the walls. The cross-sectional OCT profile acquired on the bottom of the glass vial, positioned upside down, showed two interfaces due to the different values of air/glass refractive indices (Figure 3). As from the OCT measurements (Figure 3b), the bottom of the vials were thicker than its side walls—about 0.9 mm and 0.4 mm—denoted, respectively, with yellow and blue lines in Figure 3b. We assume therefore that the laser beams cross approximately 1 mm thick glass material before impinging on the pigment powders.

**Figure 4:**
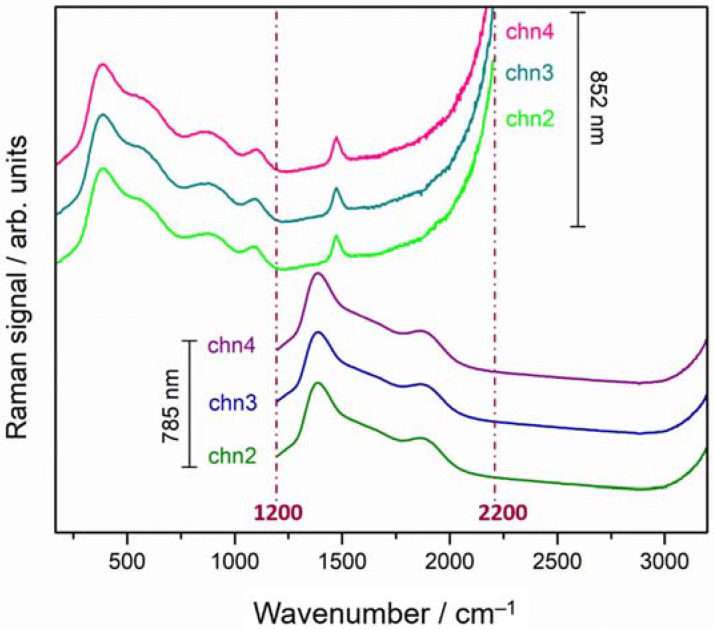
Three sequentially shifted Raman spectra acquired on the glass vials for each of the two temperature-controlled diode laser excitations (top)—852 nm (chn2–4) 170 to 2200 cm^−1^ and (bottom) —785 nm (chn2–4) 1200 to 3200 cm^−1^ (ch stands for the sequentially shifted channels as labeled in the measurement output); the vertical dashed lines define the overlapped spectral region (1200–2200 cm^−1^).

To evaluate the Raman glass contribution, the raw spectra acquired by pSSE spectrometer are shown in Figure 4. The three raw spectra obtained with the temperature-shifted excitation laser at 852 nm (covering 170 to 2200 cm^−1^) and at 785 nm (covering 1200 to 3200 cm^−1^) are characterized by intense broad bands attributed to the presence of active luminescent ion impurities in glass [20]. Typically, such broad bands completely obscure the fingerprint region of the Raman spectrum.

### 3.2. Demonstration of pSSE Raman Identification of White Material Enclosed in a Glass Vial

**Figure 5:**
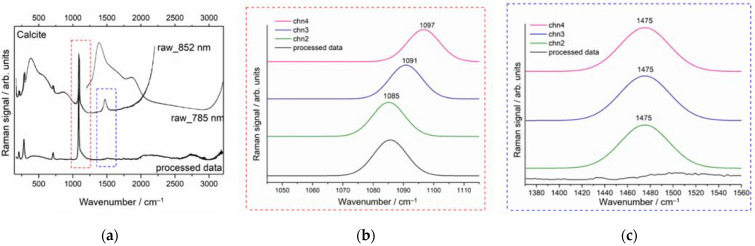
Portable Raman spectra of white substance acquired through glass attributable to (**a**) calcite (CaCO_3_), the peak at 1085 cm^−1^ assigned to ν_s_CO_3_^2−^ is marked with the red dashed rectangle while the band at 1475 cm^−1^ arising from glass container with the blue dashed rectangle; (**b**) spectral zoom on 1085 cm^−1^ shifted by 6 cm^−1^ in channels 3 (blue solid line) and 4 (pink solid line); (**c**) spectral zoom on the band at 1475 cm^−1^ remains constant in frequency in all three channels and is eliminated in SSE processed data (black solid line), providing for a background free spectrum.

Generally, the raw portable Raman spectra of pigments acquired through the glass vials were dominated by the strong fluorescence background, originating from multiple sources such as the fingerprints or other impurities present on vials or/and from the glass or examined material itself. By the way of example, Figure 5a shows the raw (top) and elaborated (bottom) spectra of white substance, acquired through a glass vial, and attributable to calcite (CaCO_3_) with a single band at 1085 cm^−1^ (ν_s_CO_3_^2−^), the δ_in-plane_CO_3_^2−^ at 711 cm^−1^ and bands below 300 cm^−1^ related to translational and rotational lattice modes. Figure 5b shows the spectral zoom on the symmetric carbonate stretch located at 1085 cm^−1^ (channel 2) that is displaced by 6 cm^−1^ in channels 3 and 4 due to the shifted laser excitation. Such band is preserved in the processed spectrum and correctly positioned at 1085 cm^−1^ (Figure 5b—black line). On the other hand, an example of efficient subtraction of the 1475 cm^−1^ band related to the glass is demonstrated in Figure 5c. The raw SSE spectra (colored lines) exhibit the same frequency band position that is subtracted in the processed spectrum (Figure 5c, black line).

The Raman data are extracted from the shifted excitation spectra by principal component analysis (PCA). The Raman spectra acquired at each unique laser excitation can be described by the matrix R:

$$R:\left(\begin{array}{ccc} r_{0,0} & \cdots & r_{0,n}\\ \vdots & \ddots & \vdots \\ r_{k,0} & \cdots & r_{k,n} \end{array}\right),$$

where the number of rows is equal to the number of excitations (K) and the number of columns is equal to the number of spectral positions (N). Each element of the matrix has two unique indices, *k* and *n*, and is represented by *r_k,n_* where the maximum value for *k* is K − 1, and the maximum value for *n* is N − 1. Using conventional matrix decomposition such as singular value decomposition (SVD), R can be related to the loadings matrix L:

R = T L^T^,

where L^T^ is the transpose of the loadings matrix and where there are K rows in L and each row corresponds to a principal component (loading vector), describing orthogonal variance in R. A characteristic of the loading matrix is that the PCs are sorted in descending order of variance. Since the principal variation of R is the change in excitation lasers when collecting the data, and since this principally only affects the Raman signal, the first PC describes the variation in the Raman signal as the excitation lasers are changed, and this is observed as a pseudo-derivative Raman spectrum. The spectral components, which are independent of small changes in excitation wavelength (e.g., fluorescence) as well as a portion of the random noise are described by the higher principal components.

### 3.3. Performance Comparison of pSSE and Bench Dispersive RS

All the materials in the wooden case were classified in eight hues (blue, yellow, red, green, violet, brown, black, and white) as show in Table 2. It summarizes their color, the sample name, and the identified material present in the glass vials and the positive or negative outcome of the measurement with the pSSE or dispersive RS instrumentations. Table A1 and Table A2 summarize the Raman bands identified in this work and their tentative assignments. 

#### 3.3.1. White and Yellow Materials in Glass Vials

**Figure 6:**
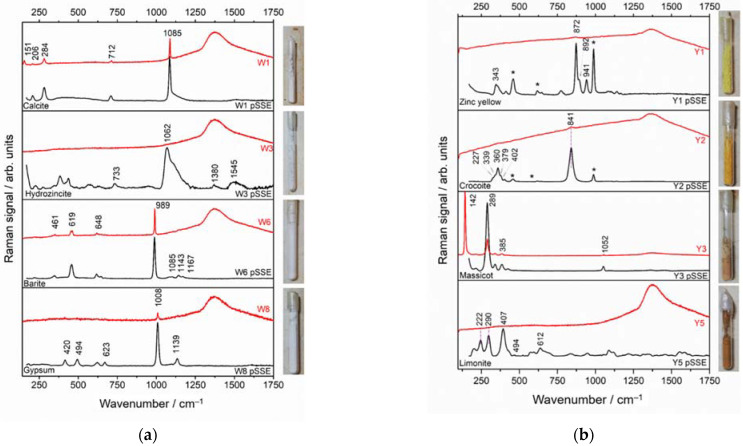
pSSE (black line) and dispersive (red line) RS of examined (**a**) white (top-to-bottom: calcite, hydrozincite, barite, gypsum); (**b**) yellow (top-to-bottom: zinc yellow, crocoite, massicot + lead white, limonite) materials. The considered spectral range is 170–1750 cm^−1^. * Indicates the bands assigned to barite.

W1 (and W2, data not shown) Raman spectra were characterized by the bands placed at 151 cm^−1^, 506 cm^−1^ and 254 cm^−1^ (lattice mode) of calcite and by 712 cm^−1^ and 1085 cm^−1^ [38]. The detection of the white pigment was enabled by both methods (pSSE and dispersive, Figure 6a). 

Hydrozincite was detected in the W3 and the W4 samples (W3 shown in Figure 6a) only by the pSSE spectra, with an intense band at 1062 cm^−1^ [39] and another band at 733 cm^−1^ [40]. The characteristic Raman bands of barite (W6, Figure 6a)—frequently used as a filler [41]—were detected by both Raman instrumentations. The spectra showed an intense band from the ν_s_SO_4_^2−^ tetrahedra at 989 cm^−1^; 461 cm^−1^, 619 cm^−1^ and 648 cm^−1^ (δSO_4_^2−^); 1085 cm^−1^, 1143 cm^−1^ and 1167 cm^−1^ (ν_as_SO_4_^2−^) [42]. W8 was characterized by the Raman bands of gypsum (Figure 6a, bottom): 1008 cm^−1^ (ν_s_SO_4_^2−^), 420 cm^−1^ and 494 cm^−1^ (δSO_4_^2−^), 1139 cm^−1^ (ν_as_SO_4_^2−^), and 623 cm^−1^ (ν_4_, SO_4_) [43]. While all the bands were observable in pSSE, only its major band placed at 1008 cm^−1^ was detected by dispersive RS. 

Seven yellow pigments were found to be chromates (zinc or lead) mixed with barite; lead (II) oxide; and hydrated iron (III) oxide-hydroxide. For the detection of chromate yellow pigments (Figure 6b), namely Y1 (ZnCrO_4_ + BaSO_4_) and Y2 (PbCrO_4_ + BaSO_4_), the pSSE Raman instrument performed very well, unveiling the most informative 774–942 cm^−1^ spectral range related to νCr-O [25,44]. Additionally, barite bands were also present. On the other hand, the spectra obtained with the benchtop instrument contained only the major peaks of Zinc Yellow (872 cm^−1^) and Crocoite (841 cm^−1^), while the barite peaks were completely covered by the glass signals. 

For the Y3 pigment, benchtop Raman acquisition provided a clear and smooth spectrum (Figure 6b), with a flat background, suggesting the examined substance with a high Raman cross-section. In addition, the extended spectral range of the benchtop instrument in the low wavenumber region provides for a confident recognition of Massicot (β-PbO) [26], with a principal band located at 142 cm^−1^ (νPb-O) [45] along with other bands at 289 and 385 cm^−1^; Y3 pigment results to be mixed with lead white (2PbCO_3_·Pb(OH)_2_ [28]), as discerned by weak band at 1052 cm^−1^. In the pSSE spectrum, the absence of the band at 142 cm^−1^ could lead to some uncertainties in pigment identification. However, being massicot the only yellow pigment with Raman lines was positioned at 289 cm^−1^ and at 385 cm^−1^, also pSSE ensures a quite confident identification. 

The Raman bands allowing the characterization of the Y5 pigment as limonite were detected only with the pSSE instrument (Figure 6b, bottom black line). The bands at 222 cm^−1^ and 494 cm^−1^ are associated with Fe–O bond, and the bands at 240 cm^−1^, 290 cm^−1^, 407 cm^−1^, and 612 cm^−1^ arise from the deformation modes of Fe oxide/hydroxide [46].

#### 3.3.2. Orange, Red, and Brown Materials in Glass Vials 

**Figure 7:**
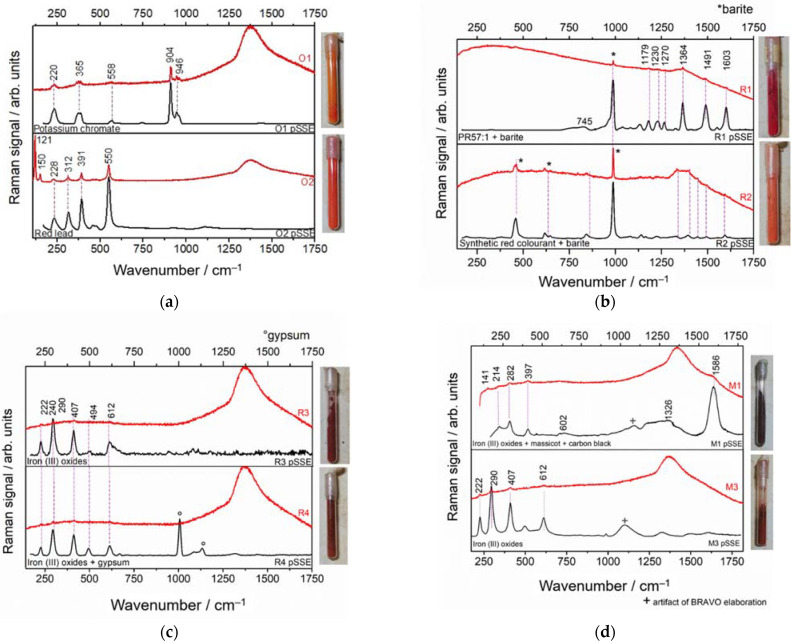
pSSE (black line) and dispersive (red line) RS of examined (**a**) orange (top-to-bottom: potassium chromate, red lead); (**b**) red (top-to-bottom: PR57:1, synthetic red colorant + barite), (**c**) red (top-to-bottom: iron(III) oxides, iron(III) oxides + gypsum); (**d**) brown (top-to-bottom: iron(III) oxides + carbon black, iron(III) oxides) materials. The considered spectral range is 170–1750 cm^−1^.

In both the benchtop and the pSSE Raman spectra of the orange O1 sample, the potassium chromate (Figure 7a) was detected by Raman bands located at: 220 cm^−1^ δ(Cr-O-Cr), 365 cm^−1^ δ(CrO_3_), 558 cm^−1^ ν_s_(Cr-O-Cr), 904 cm^−1^ ν_s_(CrO_3_), 946 cm^−1^ νs(CrO3), relative to Cr2O72− species [46]. Orange (O2-3) pigments (Figure 7a) can be identified as red lead (Pb_3_O_4_) by Raman bands at 121 cm^−1^ (vibrations of deformation of O/Pb(IV)/O angle), 150, 312, 391, and 550 cm^−1^ (vibrations of elongation of the Pb(IV)-O bond) [40]. The benchtop spectra, having the accessibility to low wavenumber spectral region (major Pb_3_O_4_ peak located at 121 cm^−1^), outperforms the pSSE instrument limited to 170 cm^−1^. 

Red and brown pigments present in the sample-case may be classified into synthetic red colorants and iron (III) oxides. In R1 (Figure 7b), the bands of the red colorant are visible in both spectra: 745, 1179, 1230, 1364, 1491, and 1603 cm^−1^ matching the PR57:1 synthetic red pigment. Instead, in R2 (Figure 7b), the bands at 1139 cm^−1^, 1445 cm^−1^, and 1595 cm^−1^ assigned, respectively, to δ_s_(C-N), ν_s_(N=N), and ν_s_(C=N) [47] are associated with a synthetic colorant from the azo group. In both cases, the characteristic bands of barite are visible. 

Red (R3, R4) and brown (M1, M3) samples are mainly iron (III) oxides (222 cm^−1^ and 494 cm^−1^ νFe-O bond [48] and (the deformation modes at 240, 290, 407, and 612 cm^−1^) [49,50]. In R3 (Figure 7b) and M3 (Figure 7c) samples, the substances are pure; instead, in R4 (Figure 7b) and M1 (Figure 7c) the iron (III) oxides are mixed with gypsum (1008 cm^−1^ and 1135 cm^−1^) and carbon black pigment (1326 cm^−1^ and 1586 cm^−1^), respectively. Only for brown pigments, the principal hematite peaks are visible in the benchtop spectra.

#### 3.3.3. Blue and Green Materials in Glass Vials 

**Figure 8:**
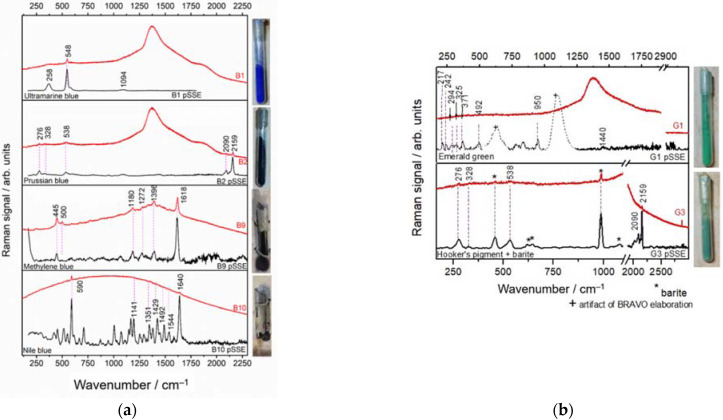
pSSE (black line) and dispersive (red line) RS of examined (**a**) blue (top-to-bottom: ultramarine blue, Prussian blue, Methylene blue, Nile blue); (**b**) green (top-to-bottom: Emerald green, Hooker’s green + barite) materials. The considered spectral range is 170–2900 cm^−1^.

Eight blue pigments (B1, B2, and B4–B9) present in sample-case were identified as: ultramarine, Prussian blue, Methylene blue, and Nile blue (Figure 8a). The Raman spectra (Figure 8a) of ultramarine (258 cm^−1^, 548 cm^−1^, and 1094 cm^−1^) [51] and Prussian blue (276 cm^−1^, 328 cm^−1^, 538 cm^−1^, 2090 cm^−1^, and 2159 cm^−1^) [52] were detectable through the glass vials by both benchtop and pSSE instruments, but it was more clearly detected in the PCA elaboration of the SSE spectra allowing for the broad bands of glass subtraction. In the spectra of Methylene blue, the characteristic bands are all detected with an excellent correspondence with both instruments: 445 and 500 cm^−1^ δ(C-N-C), 1180 cm^−1^ ν(C-N), 1272 cm^−1^, 1396 cm^−1^ (C-H), 1618 cm^−1^ ν(C–C) ring [53]. In pSSE spectra of the Nile blue (Figure 8a, bottom), the 590, 1141, 1351, 1429, 1492,1544, and 1640 cm^−1^ peaks are all well visible, instead the benchtop dispersive spectrum is dominated by an intense fluorescence background, masking the minor peaks. Only two prominent peaks appear in the spectrum at 590 and 1640 cm^−1^, related to, respectively, ring-breathing and ethylenic-stretching motions [54].

Two types of greens were identified: Emerald (G1, G2) and possibly Hooker’s green (G3), as shown in Figure 8b. Emerald green (950 and 1440 cm^−1^ of acetate group [55] and 217, 242, 294, 325, 371, 492 cm^−1^ of arseniate moieties) [56] was detected only with pSSE spectrometer, although the intensity of the peaks is very low and not all the bands related to glass were subtracted perfectly in the elaborated spectrum (marked with a plus in Figure 8b). Instead, the Raman spectra of G3 are characterized as a mixture of Prussian blue and barite, visible in both spectra, even with major clarity in the pSSE spectrum. The possible yellow component (e.g., Gamboge) was not detected.

#### 3.3.4. Violet and Black Materials in Glass Vials 

**Figure 9:**
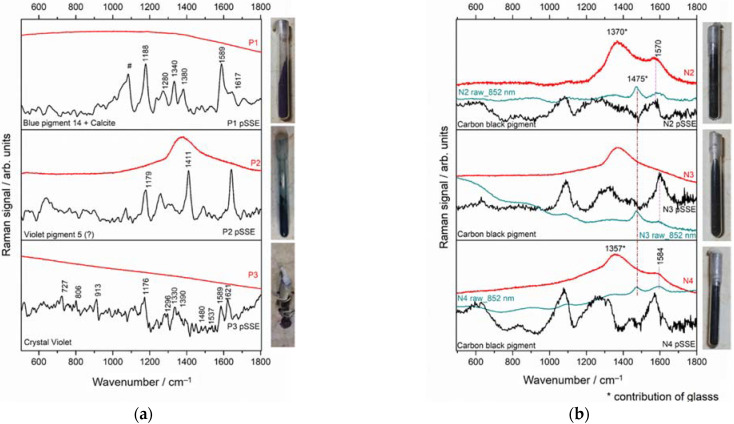
pSSE (black line) and dispersive (red line) RS of examined (**a**) violet (top-to-bottom: blue pigment + calcite, triarylmethane pigment, crystal violet); (**b**) black (carbon black) materials. The considered spectral range is 170–1800 cm^−1^. # Indicates the bands assigned to calcite.

In all the violet pigments, the Raman spectra derived from benchtop instrument are not significant (Figure 9a), instead, in pSSE measurements enabled their identification. The P1 violet pigment is as a mixture of calcite and Pigment Blue 14 (PB14) [57]. In P2, the principal peaks, located at 1179, 1411, 1642 cm^−1^, can be associated to the triarylmethane family. The signal-to-noise ratio in the P3 pSSE spectrum is low, nevertheless, the typical peaks of Crystal Violet (Basic Violet 3, BV3) (1621 cm^−1^, 1589 cm^−1^ and 1176 cm^−1^) are detectable [58].

Investigation of black substances through the vials proved challenging. Dispersive benchtop Raman spectra acquired on the black samples (N2 and 4) show the typical broad bands of carbon black pigment (Figure 9b) [59,60], even if the band at lower wavenumbers (1370 cm^−1^) has a glass contribution. In N3, no useful bands are present, but only the Raman signal of glass. The PCA pSSE elaboration proved slightly inefficient in correctly subtracting the background, creating spectral artifacts. Therefore, a careful inspection of the raw spectra is advisable (Figure 9b, green spectra). 

## 4. Discussion and Conclusions

We evaluated the performance of the portable SSE Raman spectrometer in detecting a number of colored (white, yellow, orange, red, brown, blue, green, purple, black) substances enclosed in glass vials. Comparative measurements were performed with a benchtop dispersive Raman system. Generally speaking, pSSE outperforms the standard dispersive measurements with Raman benchtop instrument exciting at 785 nm in all the instances but for the black materials. In the latter case, careful inspection of raw data must be undertaken, as the broad and the not well-defined carbon bands may be interchanged with the fluorescence interference. Good quality SSE raw spectra were obtained by properly setting the acquisition times and the number of accumulations. Indeed, the system is designed to determine and set its own operative parameters to obtain acceptable signal-to-noise ratio spectra. Typically, the proposed values range between 100–300 ms and 4–5 accumulations, but these proved insufficient for resolved and reliable spectra. The values employed (Table 1) ranged from 500–1000 ms with 5–15 accumulations. For the brown (M1, M2) and Y5 samples, as many as 150 accumulations were necessary to obtain legible data, implying acquisition times at an order of several minutes, which is an acceptable time for in situ investigation of artworks. The PCA-based algorithm has provided for the processed background free spectra that proved reliable in almost all the scenarios. Occasionally, some spectral artifacts are introduced (green, black), clearly situations in which the Raman SNR is not optimal (either due to the low Raman cross-section, or mismatch of the excitation line). 

A non-invasive approach for the identification of art materials enclosed in glass vials, without material removal, here demonstrated, enables preserving the original state of the object, and it paves the way for the examination of artwork through the protective glass shield/container. 

We have demonstrated that the pSSE Raman method is efficient in detecting most of the materials. However, the identification of black pigments through a direct application of the SSE extraction algorithm may be challenging. Indeed, the broad bands of the carbon black pigments may be subtracted because they are considered undesired backgrounds; and, furthermore, additional bands may appear just as artifacts, not containing any spectral information. We highlight the capability of the SSE to mitigate the fluorescence background providing for a clear and a linear elaborated spectrum. In the traditional benchtop Raman approach, the pigment spectra acquired through the glass container are dominated by an intense background due to the glass and fluorescence contribution rendering the spectra difficult to read. For some inorganic pigments, such as the Massicot (PbO), the benchtop instrument may outperform the portable due to accessibility to the low wavenumber region. A further research strategy will regard alternative methods of data elaboration. 

## Figures and Tables

**Table 1:** Experimental conditions (detector integration time and number of coadditions) for the powders (samples) enclosed in the glass vials for the measurements with μ-Raman benchtop and the pSSE instrument.

Sample	Detector Integration Time (s)		Number of Coadditions	
	μ-Raman	pSSE	μ-Raman	pSSE
W1,W6	10	0.5	5	10
W3	10	1	5	7
W8	10	0.5	5	5
Y1, Y2,Y3	10	0.5	5	5
Y5	10	0.5	10	150
O1, O2	10	0.5	5	5
R1	10	0.5	5	10
R2, R5	10	0.5	5	20
R3	10	0.5	5	15
M1	10	0.5	10	150
M3	10	0.5	10	100
B1,B2	10	0.5	5	5
B9	10	0.5	5	1
B10	10	0.1	5	4
G1	10	0.7	5	15
G3	10	0.5	5	10
N2, N3, N4	10	0.5	5	5
P1	10	0.7	5	15
P2, P3	10	0.5	5	5

**Table 2:** The color hue, the sample name, and the composition as identified through the glass vials. The last two columns indicate the outcome of the portable BRAVO and benchtop measurements.

Color		Sample	Identified Material	Detectability by RS	
				pSSE	μ-Raman
White	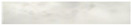	W1, W2	Calcite (CaCO_3_) [21]	Yes	Yes
	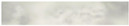	W3, W4	Hydrozincite (Zn_5_(OH)_6_(CO_3_)_2_ [22]	Yes	No
	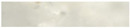	W5	Fluorescence background	No	No
	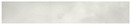	W6	Barite (BaSO_4_) [23]	Yes	Yes
	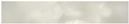	W7	Not identified	-	-
	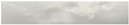	W8	Gypsum (CaSO_4_·2H_2_O) [24]	Yes	Yes
Yellow	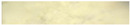	Y1	Zinc Yellow (4ZnCrO_4_·K_2_O·3H_2_O) [25] + BaSO_4_	Yes	Yes
	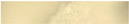	Y2, Y4, Y7	Crocoite (PbCrO_4_) [26] + BaSO_4_	Yes	Yes
	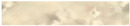	Y3, Y6	Massicot (PbO) [27] + Lead white (2PbCO_3_· Pb(OH)_2_) [28]	Yes	Yes
	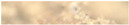	Y5	Limonite FeO(OH)_n_H_2_O [29]	Yes	No
Orange	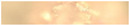	O1	Potassium Chromate (K_2_CrO) [30]	Yes	Yes
	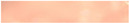	O2, O3	Red Lead (inimum) (Pb_3_O_4_) [31]	Yes	Yes
Red	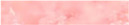	R1	PR 57:1 (C_18_H_14_CaN_2_O_6_S) + BaSO_4_	Yes	Yes
	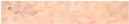	R2	BaSO_4_ + azo-colourant	Yes	Yes
	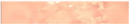	R3	Hematite (αFe_2_O_3_) [32]	Yes	Yes
	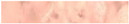	R4	Hematite + gypsum	Yes	Yes
Brown	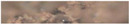	M1	Fe oxides + Massicot + carbon black	Yes	Yes
	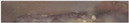	M2	Fluorescence background	No	No
	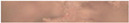	M3	Fe (III) oxides (Hematite)	Yes	Yes
	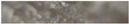	M4	Fluorescence background	No	No
Blue	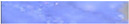	B1, B4, B5, B6	Ultramarine blue (Na_8−x_[SiAlO_4_]_6_·S_2_,S_3_,SO_4_,Cl)_2−x_) [33]	Yes	Yes
	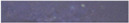	B2, B7, B8	Prussian blue (Fe_4_[Fe(CN)_6_]_3_) [34]	Yes	Yes
	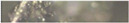	B9	Methylene blue (C_16_H_18_N_3_SCl) [35]	Yes	Yes
	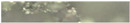	B10	Nile blue (C_20_H_20_ClN_3_O) [36]	Yes	Yes
Green	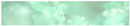	G1, G2	Emerald Green (Cu(C_2_H_3_O_2_)_2_·3Cu(AsO_2_)_2_) [37]	Yes	No
	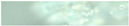	G3	Prussian Blue + BaSO_4_ + yellow pigment? (Hooker’s green?)	Yes	Yes
Black	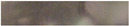	N2	Carbon black pigment	No	Yes
	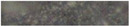	N3, N6	Carbon black pigment	No	No
	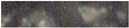	N4, N5	Carbon black pigment	No	Yes
Purple	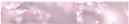	P1	PB14 (C_25_H_43_N_3_) + Calcite	Yes	No
	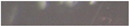	P2	Triarylmethane dye	Yes	No
	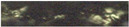	P3	Crystal Violet, BV3 (C_25_N_3_H_30_Cl)	Yes	No

## Data Availability

The data presented in this study are available on request from the corresponding author.

## Appendix A

**Table A1:** Summary of the main Raman bands and their tentative assignment of inorganic pigment present in sample-case [38,39,40,41,42,43,44,45,46,49,50,51,52,55,56,59,60]. ^•^ = Calcite, ^▼^ = Hydrozincite, ^+^ = Lead white, * = Gypsum, ^♦^ = Barite, ^∆^ = Massicot, ^†^ = Minium, ^⊗^ = Potassium Chromate, ^□^ = Zinc Yellow,^◊^ = Crocoite.

Sample	Bands (cm^−1^)	Assignment	Material
W1, W2, W3, W4, Y3, Y6, P1	1085 ^•^, 1064 ^▼^, 1052 ^+^	νCO_3_^2−^	^•^ CaCO_3_, ^▼^ (Zn_5_(OH)_6_(CO_3_)_2_, ^+^ 2PbCO_3_·Pb(OH)_2_
	711 ^•^, 733 ^▼^	δCO_3_^2−^	
	151 ^•^, 254 ^•^, 506 ^•^	CO_3_^2−^ Lattice mode	
W6, W8, Y1, Y2, Y4, Y7, G3, R1, R2, R4	1167 ^♦^, 1143 ^♦^, 1139 *, 1085 ^♦^	ν_asym_SO_4_^2−^	CaSO_4_·2H_2_O *, BaSO_4_^♦^
	1008 *, 989 ^♦^,	ν_sym_SO_4_^2−^	
	648 ^♦^, 619 ^♦^, 461 ^♦^,494 *, 420 *	δSO_4_^2−^	
Y3, Y6, O2, O3	550 ^†^, 391 ^†^, 312 ^†^, 150 ^†^, 142 ^∆^	νPb-O	^∆^ β-PbO, ^†^ Pb_3_O_4_
	121 ^†^	δPb-O	
Y2, Y5, R3, R4, M1, M3	494, 222	νFe-O	αFe_2_O_3_,
	612, 290, 240	δFe-O,	FeO(OH)_n_H_2_O, αFe_2_O_3_
	407	ν_sym_ Fe-O-Fe/-OH	
O1, Y1, Y2, Y4, Y7	946 ^⊗^	ν_asym_CrO_3_	^⊗^ K_2_CrO, ^□^ 4ZnCrO_4_·K_2_O·3H_2_O, ^◊^ PbCrO_4_,
	904 ^⊗^872 ^□^, 841 ^◊^	ν_sym_CrO_3_	
	365 ^⊗^	δCrO_3_	
	558 ^⊗^	ν_sym_Cr-O-Cr	
	220 ^⊗^	δCr-O-Cr	
G1, G2	950	νC-C	Cu(C_2_H_3_O_2_)_2_·3Cu(AsO_2_)_2_
	1440	ν−CO_2_	
	242, 217	arsenite moieties	
B1, B4, B5, B6	258	δ S_3_^−^	(Na, Ca)_8_(AlSiO_4_)_6_(SO_4_, S, Cl)_2_
	548	ν_sym_ S_3_^−^	
	1094	1ν_sym_ S_3_^−^	
B2, B7, B8	2159, 2090	νCN	Fe_4_[Fe(CN)_6_]_3_
	328, 276	δFe-C-N-Fe	
	538	νFe-C	
N2, N3, N4, N5, N6	1584-1570	C-C D2 band	Carbon black
	1370-1357	C-C D1 band	

**Table A2:** Summary of the main Raman bands and their tentative assignment of synthetic organic pigment present in sample-case [48,53,54,57,58]. ^‡^ Pigment not identified.

Sample	Bands (cm^−1^)	Assignment	Class	Material
R1, R2	745	ν_sym_(C-S)	Azo	PR 57:1 (C.I. 15850:1), ^‡^ Not identified
	1179	ν_sym_(C-N)		
	1230	ν (C–N)		
	^‡^ 1445, 1364	ν_sym_ (-N=N-)		
	1491	ν(CC)		
	1603, ^‡^ 1595	ν_sym_(C=N)		
	^‡^ 1139	δ_sym_(C-N)		
B9	445, 500	δ(C-N-C)	Triaminotriphenylmethane	AB 93 (C.I. 42780), Methylene Blue
	1180	ν_sym_(C=N)		
	1272, 1396	C-H		
	1618	ν(C–C)		
B10	590	Ring-breathing	Oxazine	BB 12 (C.I. 51180), Nile Blue
	1640	νEthylenic- motions		
P1	1188	δ(CCC)/ν(CN)	triaminotriphenylmethane basic	PB14 (C.I. 42600:1)
	1280	−		
	1340	−		
	1380	ν(C_center_C)		
	1589	ν(C-C)_ring_		
	1617	ν(C-C)_ring_		
P2, P3	1179 ^‡^, 1176	ν_sym_(CCC)/δ (CCC)breathing/δ_rocking_(CH_3_)	Triarylmethane	^‡^ Not identified, BV3 (C.I. 42555:2), Crystal violet
	1330	ν Phenyl-N		
	1411 ^‡^	Aromatic ring		
	1589	ν(C-C) ring		
	1642 ^‡^, 1621	ν_sym_(C-C)		
